# Room temperature synthesis of CdSe/CdS triangular nanoemitters and their stabilization in colloidal state and sol–gel glass†

**DOI:** 10.1039/d3ra04992b

**Published:** 2023-09-27

**Authors:** Anna Lesiak, Benoit Wagnon, Denis Chateau, Benjamin Abécassis, Stephane Parola

**Affiliations:** a Wrocław University of Science and Technology, Faculty of Chemistry Wrocław Poland anna.lesiak@pwr.edu.pl; b École Normale Supérieure de Lyon, Chemistry Laboratory, CNRS, University Lyon 1, UMR 5182 Lyon France stephane.parola@ens-lyon.fr

## Abstract

Heterostructured cadmium-based core–shell nanoparticles (NPs) are the subject of research because of not only fundamental scientific advances but also a range of technological applications. To increase the range of applications of nanoparticles, it is possible to immobilise them in sol–gel glass that can be easily manufactured and shaped, keeping the properties of the dispersed particles. This allows the creation of new bulk optical materials with tailored properties, opening up opportunities for various technological applications such as lighting or sensing. Herein we report the synthesis of core–shell CdSe/CdS triangular-shaped nanoparticles under an atmosphere of oxygen and at room temperature. A detailed characterisation of the obtained NPs was carried out. The interesting effect of the gelling agent (tetra-*n*-butylammonium fluoride) on the triangular nanoparticles in solution and the stability of the emission properties over time was investigated. Sol–gel glasses with entrapped triangular NPs were prepared, and their photoluminescence properties were compared with those obtained in colloidal solutions.

## Abstract 2

Heterostructured cadmium-based core–shell nanoparticles (NPs) are the subject of research because of not only fundamental scientific advances but also a range of technological applications.

## Graphical abstract

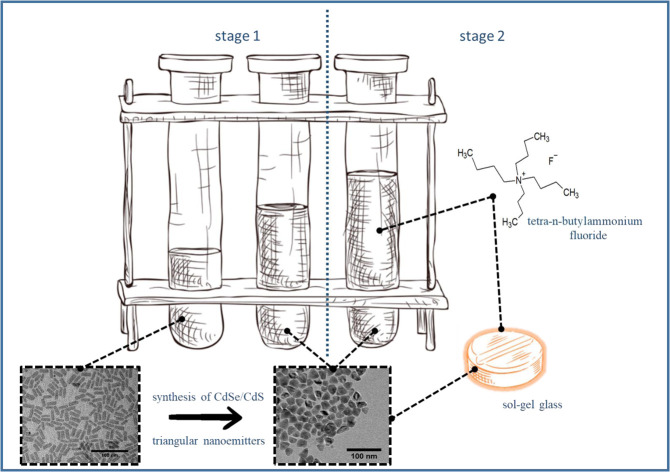

## 1. Introduction

Cadmium-based nanoparticles (NPs) can be synthesised in a wide variety of geometries, regular and irregular in shape.^1–5^ Anisotropic structures, such as rods and triangular shapes, have higher chemical potentials than isotropic spherical nanoparticles (if they have the same volume) due to their higher surface energy.^6^ Usually, the most common methods for the synthesis of high-quality cadmium-based nanoparticles are high-temperature thermal reactions in organic solvents, which is considered a key factor influencing the shape control process during the reaction.^7,8^ Despite this, the triangular structures most commonly described in the literature are usually only core structures, such as CdSe or CdS nanoparticles. For instance, Chen W. *et al.*^6^ reported the solvothermal synthesis of triangular wurtzite CdS NPs, reactions were conducted at high temperatures (140 °C, 180 °C, and 207 °C). As a result, they obtained nanoparticles shaped like triangles, but which had flat structures. Jin B. *et al.*^9^ presented research on the initially self-limited epitaxial growth of ultrathin non-layered CdS flakes with triangular shape prepared by the physical vapour deposition method. The reaction carried out used an oxygen-free environment and samples were heated to 930 °C. Cheng Y. *et al.* also performed synthesis under high temperature conditions (220 °C for 10 h in an oven), and prepared a shape-controlled synthesis of monodisperse CdS wurtzite with pyramidal geometry.^10^

While less prevalent in occurrence, the literature also documents the synthesis of core–shell nanoparticles featuring a triangular morphology. For instance, Pun A. B. *et al.*^11^ demonstrated the fabrication of cadmium-based core–shell nanoparticles with a triangular configuration, employing elevated reaction temperatures (110–180 °C). In their method, the shell growth was achieved on a pre-existing triangular core structure.

The immobilisation of nanoparticles in glass offers a versatile approach to the creation of optical materials with tailored properties, opening up opportunities for various innovation in optics (*e.g.* optical devices, energy conversion and storage, sensing and biosensing).^12–16^ Nanoparticles can be entrapped in a solid matrix through a doping process^17^ or as a simultaneous matrix and NPs synthesis process. For example, Algradee *et al.*^18^ carried out a parallel synthesis of nanoparticles and matrix, obtaining embedded CdS nanocrystals (rounded shape and approximate diameters in the range of 2 to 4.5 nm) in a phosphate glass matrix. An important route to incorporate nanoparticles into hybrid silica-based matrices is the sol–gel process. It operates at low temperature and allows the preservation of the optical properties of the introduced nanoparticles.^19^

In this study, we have demonstrated an approach to the synthesis of triangular CdSe/CdS core–shell nanoparticles at room temperature. A comprehensive characterisation of the obtained nanoparticles was carried out. We also investigated the effect of the gelling agent (tetra-*n*-butylammonium fluoride) on the behaviour of the triangular nanoparticles in solution, and investigated the stability of their emission properties over time. By entrapping obtained triangular nanoparticles in sol–gel glass, we prepared solid materials and evaluated their photoluminescence properties, comparing them with those observed in colloidal solutions.

## 2. Experimental section

### 2.1. Materials

Cadmium chloride (CdCl_2_, 99.999%), octylamine (99%), thioacetamide (TAA, 99.0%), ethanol (99.8%), chloroform (99.8%), tetrahydrofuran (THF, 99.0%), tetra-*n*-butylammonium fluoride (TBAF, 1.0 M in THF), sodium oleate (82.0%), methanol (99.0%), selenium powder (99.5%), cadmium acetate dihydrate (98.0%) and oleic acid (90.0%) were purchased from Sigma-Aldrich. 1-Octadecene (ODE, 90.0%) and hexane (90.0%) were supplied by Fisher-Agros. Acetone (99.8%) was purchased from Fisher Chemical. Ethyl acetate (99%) and cadmium nitrate tetrahydrate (98.5%) were supplied by Fisher-Alfa Aesar. Methyltriethoxysilane (MTEOS, 98.0%) and tetraethyl orthosilicate (TEOS, 98.0%) were purchased from ABCR.

### 2.2. Preparation of Cd-based nanoparticles

#### 2.2.1. Synthesis of CdSe nanoplatelets

The synthesis of CdSe nanoplatelets (NPLs) was performed according to the procedure presented by Ithurria *et al.* (including preparation of cadmium oleate).^20,21^ Briefly, 808 mg of cadmium oleate, 27 mg of selenium powder and 25 ml of ODE were inserted into 50 ml three-neck round bottom flask. This mixture was degassed under vacuum for 1 h. The temperature was then raised to 240 °C under argon flow. At 205 °C, when the colour was yellow-orange, 280 mg of cadmium acetate dihydrate were quickly injected. The colour of the mixture changed from yellow-orange to deep red. Annealing at 240 °C was carried out for 10 min before injecting 1 ml of oleic acid and cooling down the flask to room temperature using a water bath.

The nanoplatelets were separated from the remaining reactants and quantum dots by centrifuging the crude for 10 min at 6000 rpm in the presence of 2.5 ml of acetone. The solid was removed and the supernatant was centrifuged at 6000 rpm for 10 min. The last step was reproduced once. 5 ml of acetone was added to the remaining of NPLs solution before centrifuging at 6000 rpm for 10 min. Finally, the solid obtained was resuspended in 10 ml of hexane.

#### 2.2.2. Synthesis of CdS shell and change of shape

##### 2.2.2.1. Preparation of CdCl_2_/Cd(OL)_2_

The mixture of CdCl_2_/Cd(OL)_2_ were synthesised following a procedure of Christodoulou *et al.*^22^ Briefly, 20 mg of CdCl_2_ and 420 mg of Cd(OL)_2_ (powder) dispersed in 10 ml of oleic acid were heated for 15 min at 200 °C under argon flow and then sonicated for 30 min at room temperature to obtain a white suspension.

##### 2.2.2.2. Triangular shell growth

Synthesis of CdS shell was adapted from the procedure presented by Woznica *et al.*^23^ 0.5 ml of CdSe nanoplatelets solution (in hexane) was mixed with 2 ml of chloroform, 20 mg of TAA, and 200 μl of octylamine in a 4 ml flask. The solution was placed in the ultrasonic cleaner until the TAA was dissolved. After 2 h, 60 μl of CdCl_2_/Cd(OL)_2_ was added and the mixture was left to mix for 72 h at room temperature. The reaction was conducted in an ambient atmosphere without inert gas or vacuum connection. To precipitate NPs, the solution was transferred to centrifuge tubes and 2 ml of ethanol was added. After 10 min of centrifuging at 6000 rpm, the NPs were precipitated. The supernatant was discarded and the precipitate was dissolved in THF. The precipitation/dissolution procedure was repeated three times before further application. The resulting colloidal solution of CdSe/CdS triangles had a concentration of 5.55 mg ml^−1^.

### 2.3. Nanoparticles characterisation

Energy-dispersive X-ray spectroscopy (EDS) and transmission electron microscopy (TEM) images were obtained with a JEOL 2100F equipped with a Gatan ultrascan 1000 camera operating at 200 kV microscope. Samples were prepared by evaporation of diluted solutions of purified nanoparticles on carbon-coated copper grids. Absorbance spectra (ABS) were measured on a JASCO V-770 spectrophotometer. The emission was collected by a spectrofluorimeter FLUOROLOG-3 (by Horiba) using excitation wavelength 400 nm. X-ray diffraction analysis (XRD) was performed with an Empyrean X-ray diffractometer (Malvern Panalytical) using CuKα_1,2_ radiation in the Bragg–Brentano. Small-angle X-ray scattering (SAXS) measurements were carried out on a XENOCS Xeuss 3.0 instrument. Dynamic light scattering (DLS) measurements were prepared by MALVERN ZETASIZER NANO-ZS device.

### 2.4. Preparation of colloidal suspensions for spectroscopy

226 μl of previously prepared nanoparticles were used to prepare the CdSe/CdS NPs solutions and filled with THF to a volume of 2 ml. To study the effect of the tetra-*n*-butylammonium fluoride (TBAF) on the optical properties of CdSe/CdS nanoparticles, 226 μl of NPs solution, 40 μl of TBAF were used, and the volume was supplemented to 2 ml by THF.

### 2.5. Preparation of sol–gel glasses

In order to obtain a solid glass matrix, it is necessary to use a gelling agent to promote the solidification process. Many gelling systems that can form monolithic glasses are applied to the formation of these materials.^24,25^ As gelling agents can be used amine derivatives of silanes (*e.g.* aminopropyltriethoxysilane (APTES),^26,27^*N*,*N*-dimethylaminopropyltrimethoxysilane^17^) or tetra-*n*-butylammonium fluoride (TBAF).^28^ In our case, TBAF was selected for the control of the gelation step. A suspension of CdSe/CdS NPs (30 μl) in THF was introduced into a MTEOS/TEOS sol (0.67 g), prepared using an adapted procedure from previously reported method,^19,29^ and filled to a volume of approximately 1 ml of THF. Next, 40 μl of gelling agent (TBAF) in THF was added to the mixture in the mould. The mould was closed with a cap with a small hole allowing controlled evaporation of the solvent, and gently stirred by hand for 30 seconds before keeping at room temperature until gelation occurred (usually about 20–30 min). The gelled material was placed in an oven at 45 °C for 3 days to obtain the final solid material.

## 3. Results and discussion

### 3.1. Nanoparticles

**Fig. 1:**
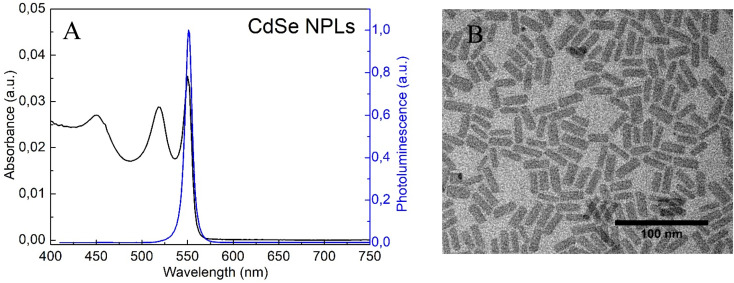
(A) Absorbance and emission spectra and (B) TEM image of NPLs before surface modification.

The absorbance and photoluminescence (PL) spectra of CdSe nanoplatelets are shown in Fig. 1A. The sample showed the formation of NPLs with thicknesses of 5.5 monolayers and maximum emission at 550 nm as previously reported.^22^ The structure of the NPLs obtained was confirmed by TEM measurements (Fig. 1B). The morphological presentation of the NPLs showed that they are flat sheets with average sizes of 22 nm in length and 8.4 nm in width.

In order to perform surface modification of NPLs, thioacetamide was introduced into the system as a source of sulphur. Based on the fact that the shape and crystalline structure of Cd-based nanoplatelets are defined by surface ligand applied during synthesis, any post-synthesis modification of the nanoplates surface should be carried out very carefully.^30^ The surface energy change implied by the exchange of the ligand or the formation of the shell may cause the degradation and/or transformation of the nanoplates into quantum dots.^31^

During the reaction, nanoplatelets rearranged into CdSe quantum dots and has begun the growth of the CdS shell (Fig. S1†). The absorbance properties of the nanoparticles show a dependence on the reaction time with TAA. As the reaction time increases, a shift in absorbance toward longer wavelengths is apparent. This may indicate growth and accumulation of nanoparticles, leading to higher light absorption capacities. However, with longer reaction time the absorbance may stabilise or even decrease, indicating potential aggregation or saturation effects.^32,33^ Examination of the photoluminescence properties as a function of reaction time reveals interesting trends. Initially, shorter reaction times result in less efficient photoluminescence signals, because the NPs may not have reached their optimal emission state. As the reaction time increases, the photoluminescence intensity increases, indicating improved NPs quality and increased emission efficiency. However, an excessively prolonged reaction time may result in quenching effects that reduce photoluminescence. On the basis of, it can be assumed that the reaction time with TAA influences the growth of the CdS shell (observed red shift in the absorbance and emission spectra), but allows only spherical structures to be obtained. Therefore, a small amount of CdCl_2_/Cd(OL)_2_ was introduced into the reaction mixture to initiate growth in a different shape.^22,34^

**Fig. 2:**
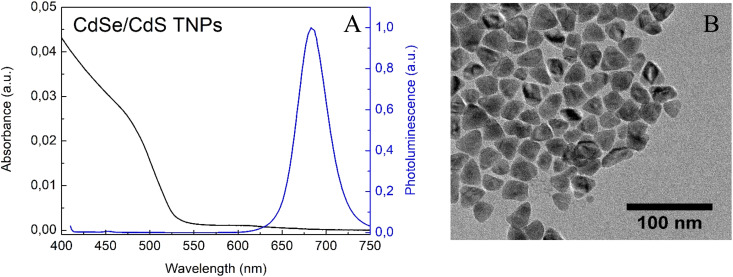
(A) Absorbance and emission spectra and (B) TEM image of triangular nanoparticles.

After 72 h of adding CdCl_2_/Cd(OL)_2_, reaction was stopped and the characterisation of the obtained nanoparticles was carried out. NPs with maximum emission at 680 nm and triangular shape were obtained (Fig. 2A and B). Since the quantum yield of the obtained CdSe/CdS nanoparticles was 52%, it can be deduced that the state defects remained localised in the CdSe core, which were formed during the decomposition of the nanoplatelets.^35^

To demonstrate the chemical composition of the NPs obtained, energy-dispersive X-ray spectroscopy (EDX) was used. Elemental analysis of the material showed that NPs had CdSe core and CdS shell, with a gradient in thicknesses. In fact, the coating is thicker on the edges than on the faces of the nanoparticle (Fig. S2†). The low % Se in EDX can be explained by the heterogeneity of the CdS coating.^36^ It can be assumed that the formation of an additional atomic layer has covered the core, which consists of Se and is difficult to detect accurately.

**Fig. 3:**
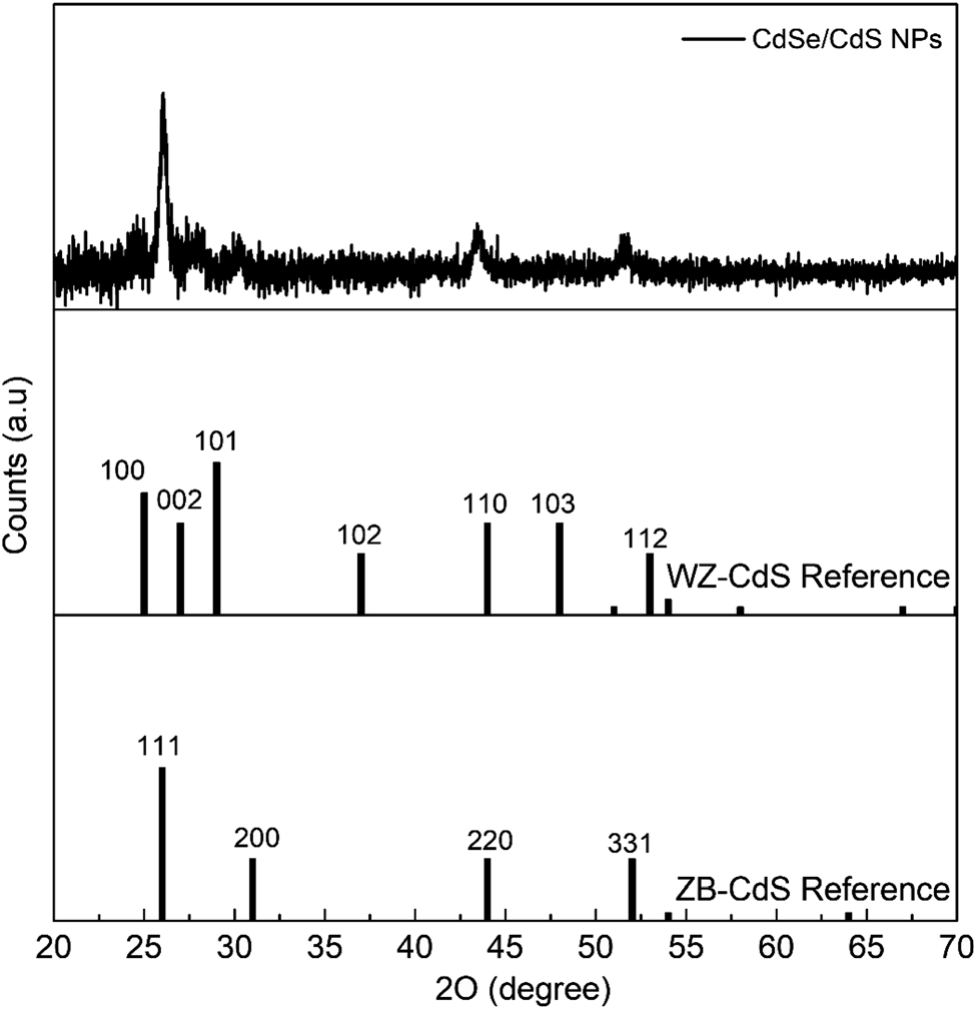
X-ray diffraction patterns for triangular CdSe/CdS NPs grown in the crystal structure of cubic zinc-blende. The corresponding diffraction references of bulk WZ- and ZB-CdS are given below.

The X-ray diffraction (XRD) pattern for the synthesised triangular nanoparticles and, based on the EDS results, the corresponding bulk WZ- and ZB-CdS diffraction references are shown in Fig. 3. The interplanar spacings calculated from the 2-theta positions of the peaks in the XRD pattern (JCPDS card no. 89-0440) correspond to the (111), (220), and (331) planes, which are characteristic of the cubic phase of CdS.^37,38^ However, small shifts of the peaks from the reference values were observed. This may be a consequence of the small scale of the crystal relative to the bulk crystal, or may be due to the strain imposed on the crystal lattice by the core–shell lattice mismatch.^36^ In addition, a SAXS analysis was also performed (Fig. S3†). Based on the bell shaped curve, it can be said that the NPs studied are close to spherical and therefore three-dimensional structures.^39,40^

**Fig. 4:**
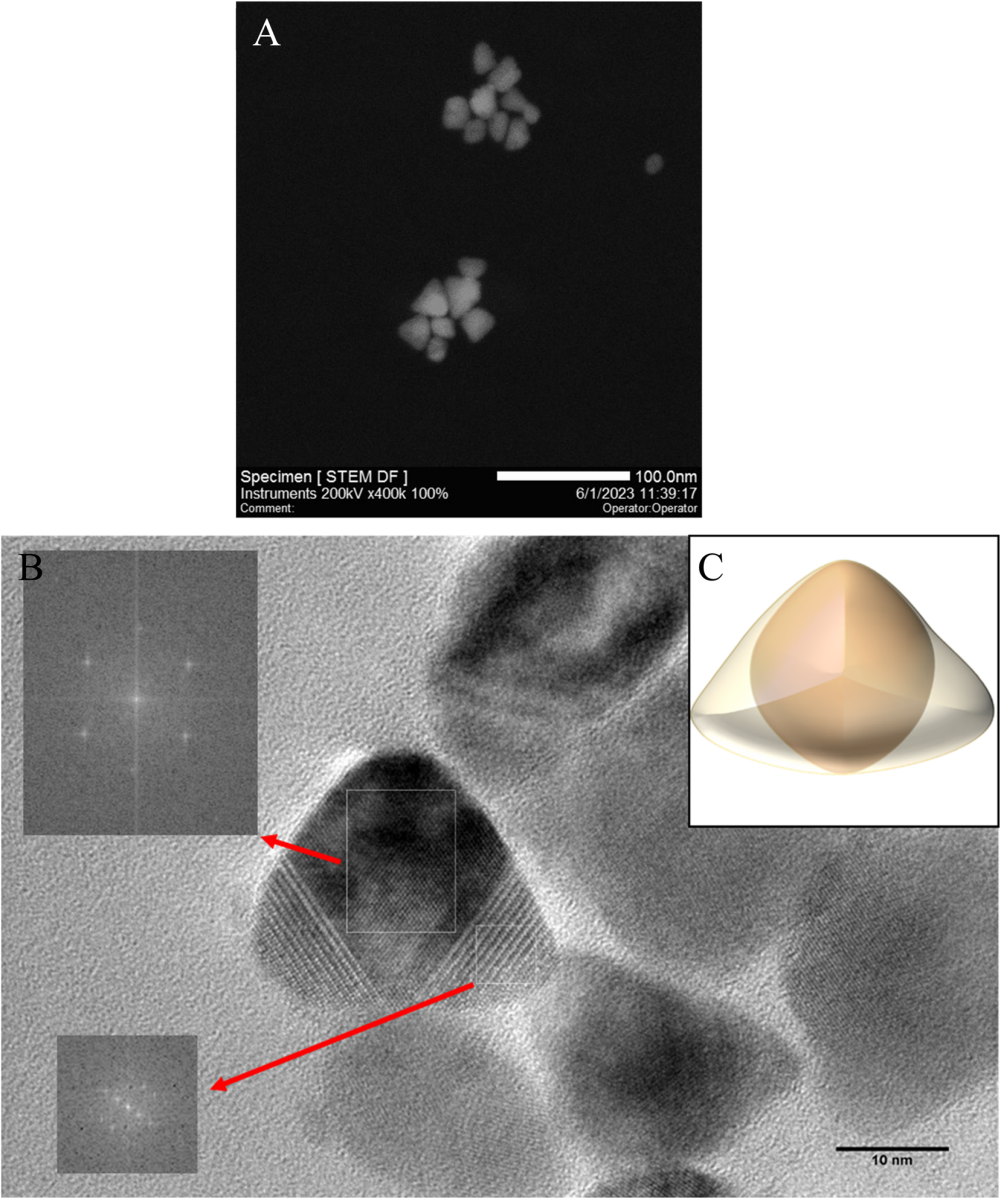
CdSe/CdS nanoparticles illustrated by (A) STEM and (B) HRTEM pictures and the corresponding fast Fourier transform (FFT) patterns; (C) visualisation of the obtained nanoparticles on the basis of the results obtained.

To confirm the three-dimensional structures of the resulting triangular nanoparticles, HRTEM and STEM measurements were carried out (Fig. 4). The contrast of NPs obtained suggests that the triangular nanoparticles have a three-dimensional structure and therefore are tetrahedral with rounded vertices.^38,41^ It can suggested a larger surface energy and a higher reactivity of the obtained NPs.^3^ The average size of the regular tetrahedrons was determined by lengths the edge and is 27 nm ± 4 nm. NPs were found to be highly crystalline and clearly exhibit a cubic morphology, as well as cubic symmetry at the atomic scale.^36^ Based on the results obtained, a proposed 3D visualisation of the nanoparticle has been prepared (Fig. 4C). The extended spherical CdSe core is assumed to be covered by a triangular CdS shell. The triangular CdSe/CdS nanoparticles prepared can exist stably and retain emission properties for several months in nonpolar solvents such as THF, hexane and toluene.

### 3.2. Effect of tetra-*n*-butylammonium fluoride on nanoemitters in colloidal suspension

**Fig. 5:**
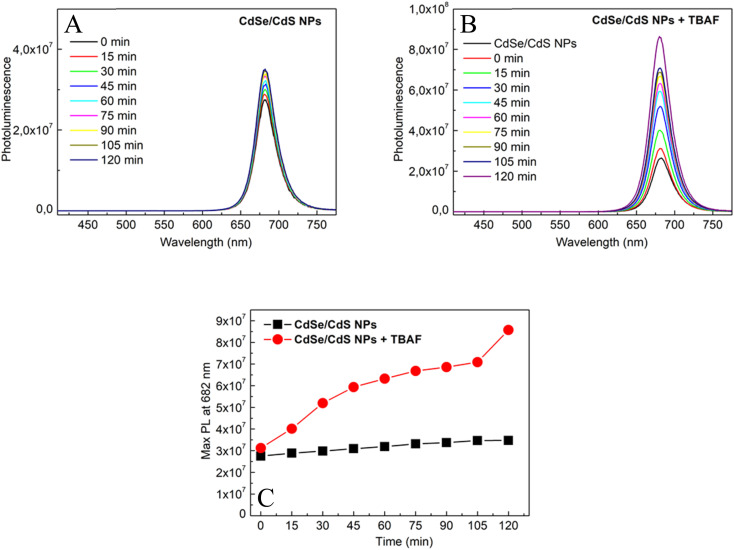
Emission spectra for (A) CdSe/CdS NPs and (B) CdSe/CdS + TBAF in time 0–120 min; (C) maximum intensity of photoluminescence for CdSe/CdS NPs in time before (black) and after addition of TBAF (red).

The effect of TBAF on the photoluminescence of CdSe/CdS nanoparticles in solution was investigated. Fig. 5 presents a linear increase in photoluminescence over time (0–120 minutes) for only CdSe/CdS NPs and CdSe/CdS NPs after the addition of TBAF. A significantly stronger growth in NPs emission was observed when TBAF was added as compared to the initial nanoparticles. These results are explained by the presence of F^−^ anions in TBAF. Negative fluorine ions interact with the surface of cadmium NPs, more precisely with the broken bonds of surface atoms (atoms with lower coordination number).^42^ As a result of these interactions, surface passivation (displacement of carrier trapping levels from the energy gap) could occur by F^−^ ions, thus reducing the possibility of nonradiative relaxation. To confirm this theory, an experiment was carried out with low-emission nanoparticles and TBAF over time (Fig. S4†), and an increase in emissions was observed. Enhancement of photoluminescence may suggest the formation of an additional shell of F^−^ ions around a single nanoparticle.^19,43^ Due to the increase in emission, we assume that there is an additional TBAF layer on the surface of the NPs, rather than ligand exchange (which usually causes defects in the states and a decrease in photoluminescence).^44–46^ However, based on other observations (Fig. S5†), it is also possible to suggest an increase in emission due to the aggregation of NPs, with the anchored TBAF between them interacting not only with the surface of one NP, but also with others. SAXS was measured after adding TBAF in two volumes: the same as during the entire experiment (40 μl) and in double volume (80 μl) (Fig. S6†). The slope and slenderness of the obtained curve, after the addition of TBAF, indicates an increase in the size of the nanoparticle.^47^ Additionally, a DLS study was also conducted, which showed reduced colloidal stability in solution 2 hours after the addition of TBAF (Fig. S7†). The results obtained may suggest that NPs aggregates have lower surface defects and thus higher emission over time.

### 3.3. Glass containing CdSe/CdS NPs: comparison of the optical properties of glass and colloidal suspensions

**Fig. 6:**
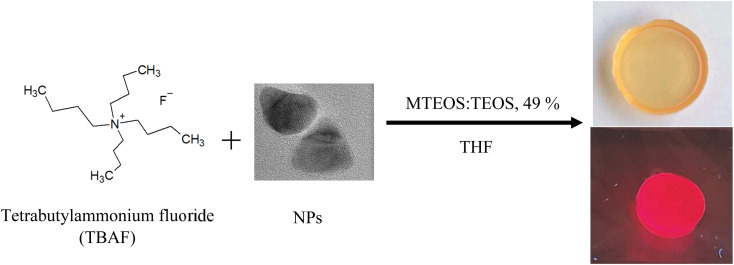
Dried glass with CdSe/CdS nanoparticles prepared using TBAF as gelling agents.

Sol–gel luminescent glasses were prepared by a mixture of hydrolysed in acidic medium methyltriethoxysilane and tetraethyl orthosilicate sol in tetrahydrofuran (MTEOS:TEOS, 49%, THF) and addition of CdSe/CdS nanoparticles in THF. To introduce a fast condensation of the sol, TBAF was added as a gelling agent. During the gelation process, an increase in the emission of NPs has been noticed similar to that observed in solution. The enhancement in the emission of CdSe/CdS NPs was observed after drying (Fig. 6).

**Fig. 7:**
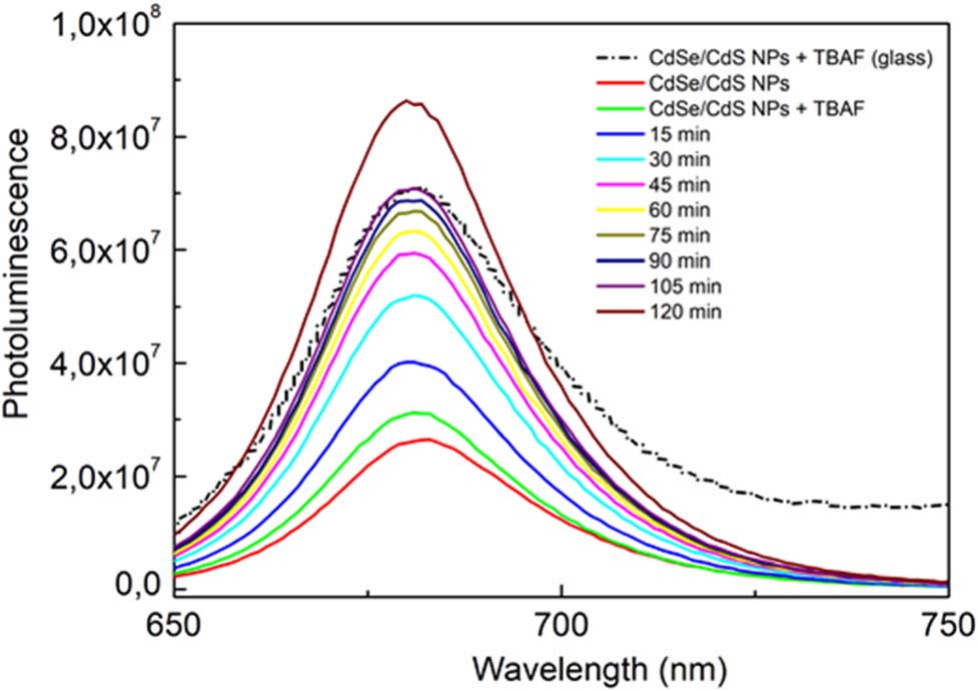
Changes in emission over time for CdSe/CdS nanoparticles after the addition of TBAF in colloidal suspension (continuous lines) and glass containing the same composition (dashed line).

In colloidal suspensions, it was possible to observe changes in emission over time. In the case of glasses, measurements could only be taken after they had solidified (the sol was a dense mixture, strongly scattering light, and the solidification process was quite rapid). Fig. 7 shows the results of measurements taken over time for CdSe/CdS NPs after the addition of TBAF to colloidal suspension and glass containing the same composition. The results show that the emission in the glass was stopped (during the solidification process) at a value close to that obtained after 105 min of measurement for the colloidal suspension. The solidification process started about 15 min after the addition of TBAF to the sol, but it took an additional 3 days at 40 °C to obtain the final material, when the solid matrix was completely dried. The nanoparticles entrapped in the glass matrix finally showed good photoluminescence properties.

**Fig. 8:**
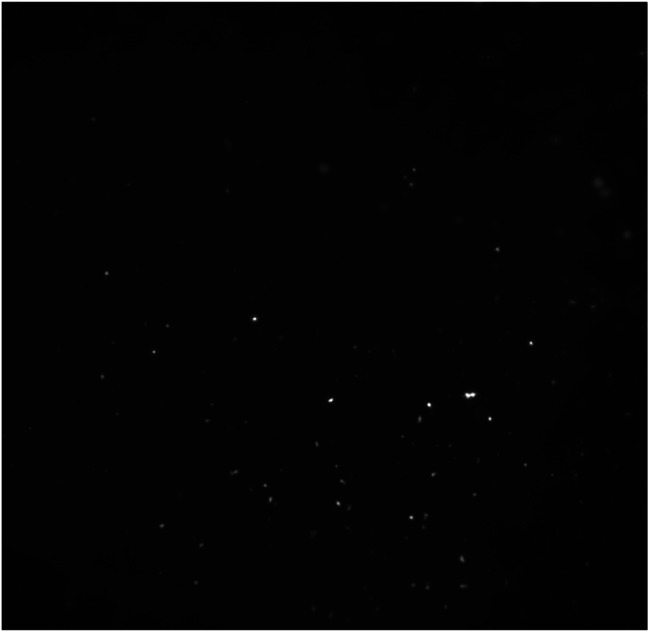
Fluorescence microscope image of sol–gel glass with CdSe/CdS nanoparticles.

As mentioned above, in colloidal solution NPs are exposed to interactions that can cause them to aggregate over time. Incorporating NPs in a solid matrix allows to protect their optical properties at a stable level (Fig. S8†).^17^ Due to the rapid solidification process, the entrapment of NPs in the solid matrix results in a rather uniform distribution of NPs in the material (Fig. 8). Moreover, the hybrid matrix obtained exhibits high transparency in the visible range, which is due to the good dispersion of NPs.^48^

## 4. Conclusions

This paper presents a method for the synthesis of CdSe/CdS core–shell nanoparticles with a triangular shape. After careful morphological analysis, it was determined that the three-dimensional triangles had a zinc blend structure. Tetra-*n*-butylammonium fluoride has been shown to increase nanoparticle emission in both colloidal solution and sol–gel glass where it acts both as a surface modifier and as a gelation catalyst. The encapsulation of CdSe/CdS nanoparticles in sol–gel glass allows the nanoparticle emission to remain stable over time.

## Author contributions

The manuscript was written through contributions of all authors. All authors have given approval to the final version of the manuscript.

## Conflicts of interest

There are no conflicts to declare.

## Supplementary Material

RA-013-D3RA04992B-s001
