# Supplementary material for: Room temperature synthesis of CdSe/CdS triangular nanoemitters and their stabilization in colloidal state and sol–gel glass
Source: RSC Adv. 2023 Sep 27;13(41):28407–15. doi: 10.1039/d3ra04992b (PMC10523092; doi:10.1039/d3ra04992b)
Supplement: RA-013-D3RA04992B-s001 [file RA-013-D3RA04992B-s001.pdf]

Electronic Supplementary Material (ESI) for RSC Advances.
This journal is © The Royal Society of Chemistry 2023

Supporting Information: The impact of reaction time using TAA on the optical properties of nanoparticles (absorbance and photoluminescence) and TEM image of intermediate product of surface modification after 2h; An Energy-dispersive X-ray spectroscopy (EDX) made in the centre and on the edges of the CdSe/CdS nanoparticle; SAXS measurements for output sample (nanoplateles) and obtained nanoparticles (triangles); Emission comparison for CdSe/CdS nanoparticles detailed described in the paper and CdSe/CdS nanoparticles with low emission; Changes in the emission of low photoluminescence CdSe/CdS nanoparticles after addition of TBAF over time; Absorbance and emission changes of triangular CdSe/CdS nanoparticles after increasing amounts of TBAF; SAXS measurements for CdSe/CdS triangles and after addition of 40 µl and 80 µl of TBAF; DLS results for CdSe/CdS triangles and 2h after adding of TBAF; Photoluminescence measurements for nanoparticles in sol-gel glass after preparation and after 9 months of storage.

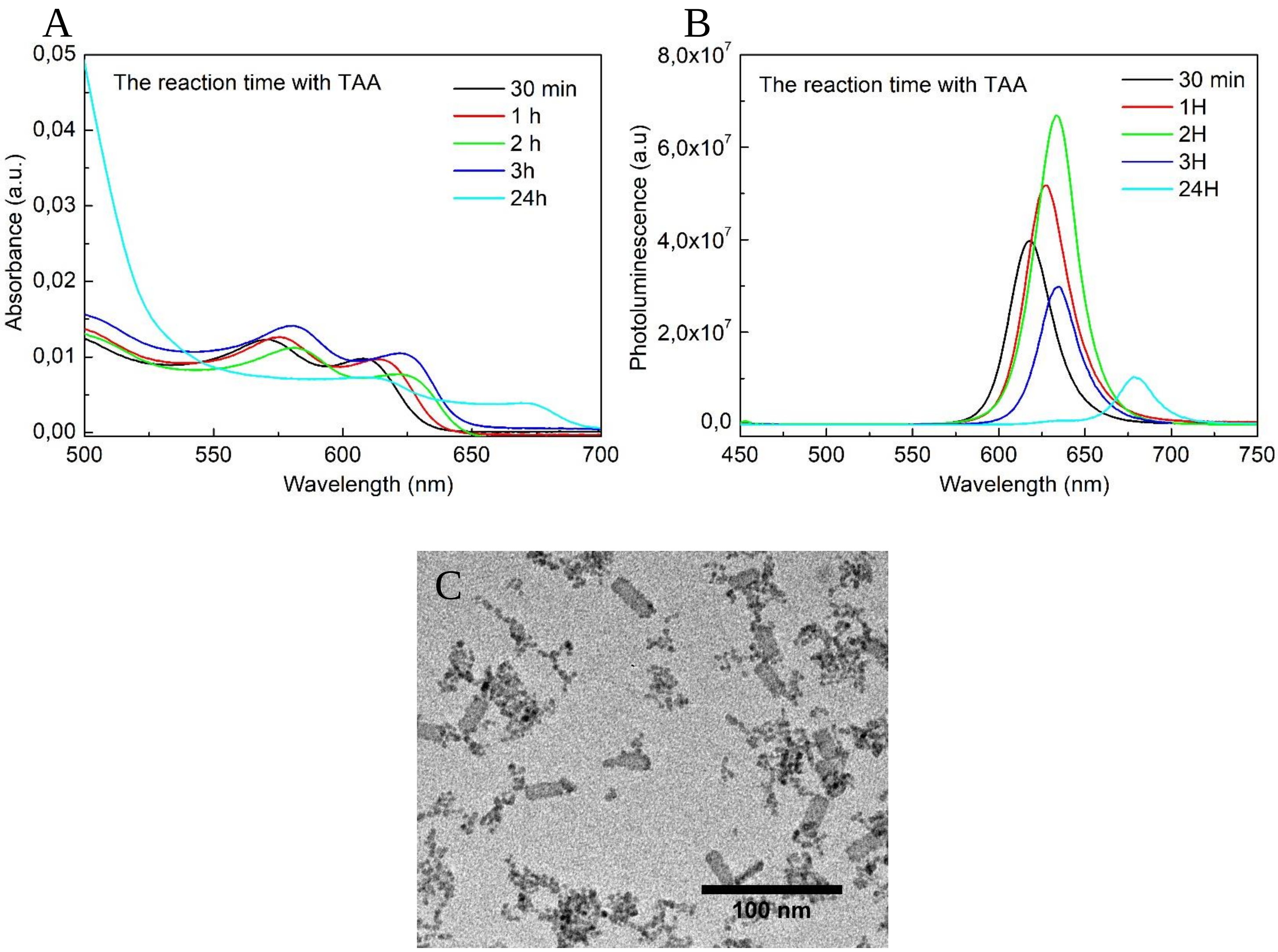

Figure S1. Effect of reaction time with TAA on A) absorbance and B) photoluminescence optical properties of nanoparticles C) TEM image of the degradation of NPLs to spherical structures (intermediate product of surface modification after 2 h).

A

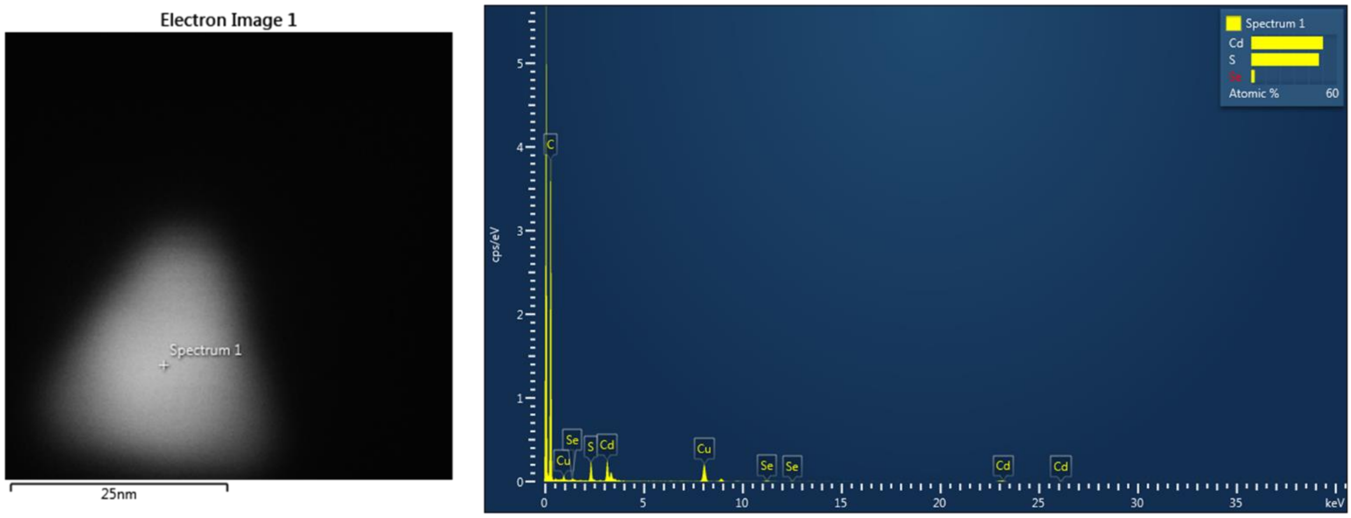

Element	Line Type	k Factor	k Factor type	Absorption Correction	Wt%	Wt% Sigma	Atomic %
S	K series	1.006	Theoretical	1.00	20.71	0.76	47.48
Se	L series	1.719	Theoretical	1.00	2.43	0.72	2.27
Cd	L series	1.887	Theoretical	1.00	76.85	0.95	50.25
Total:					100.00		100.00

B

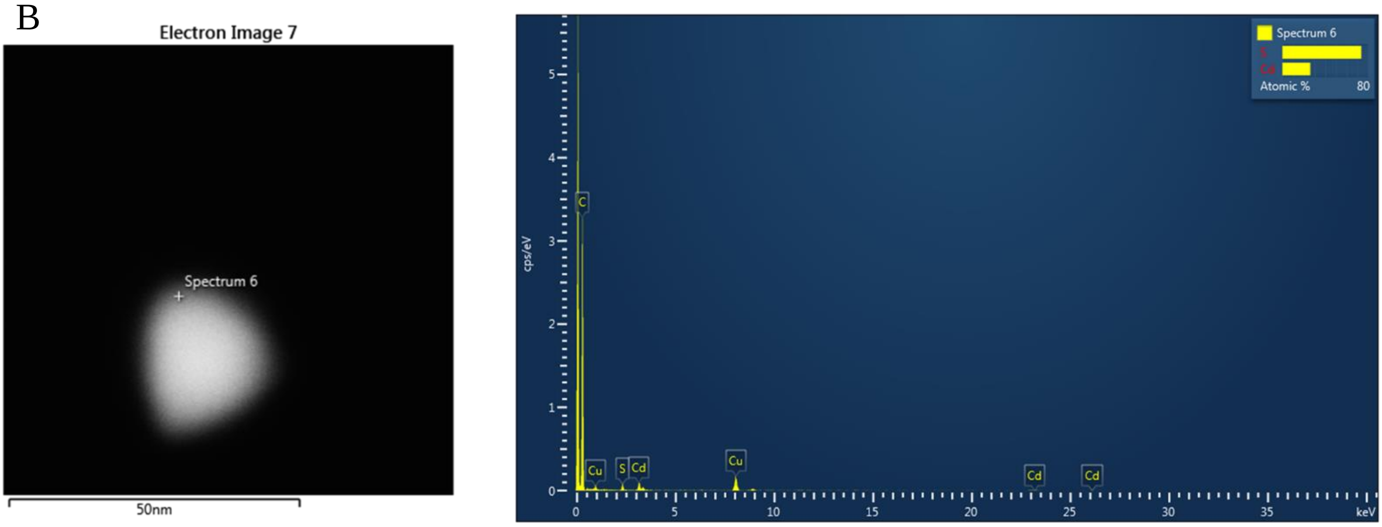

Element	Line Type	k Factor	k Factor type	Absorption Correction	Wt%	Wt% Sigma	Atomic %
S	K series	1.006	Theoretical	0.86	44.63	15.18	73.86
Cd	K series	12.546	Theoretical	0.74	55.37	15.18	26.14
Total:					100.00		100.00

Figure S2. Energy-dispersive X-ray spectroscopy (EDX) made A) in the centre and B) on the edges of the CdSe/CdS nanoparticle.

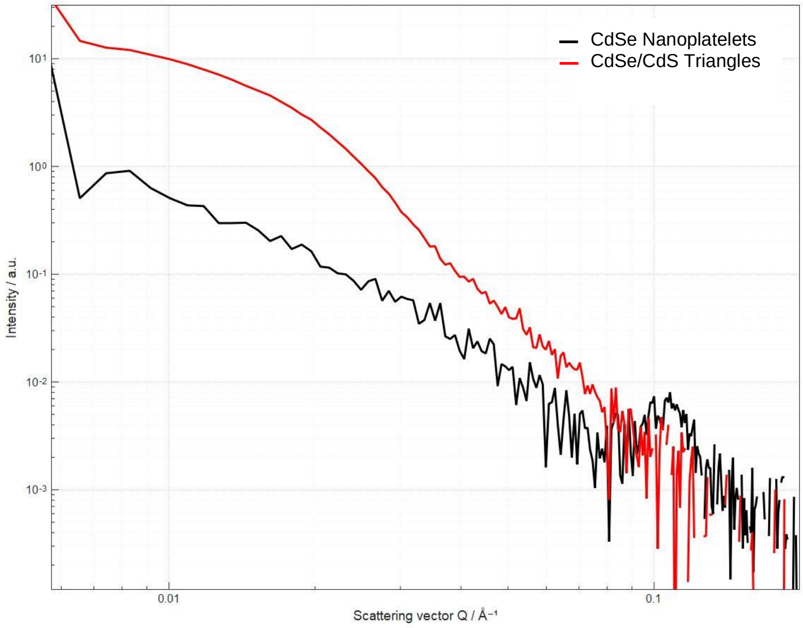

Figure S3. SAXS measurements for output sample (nanoplateles) and obtained nanoparticles (triangles).

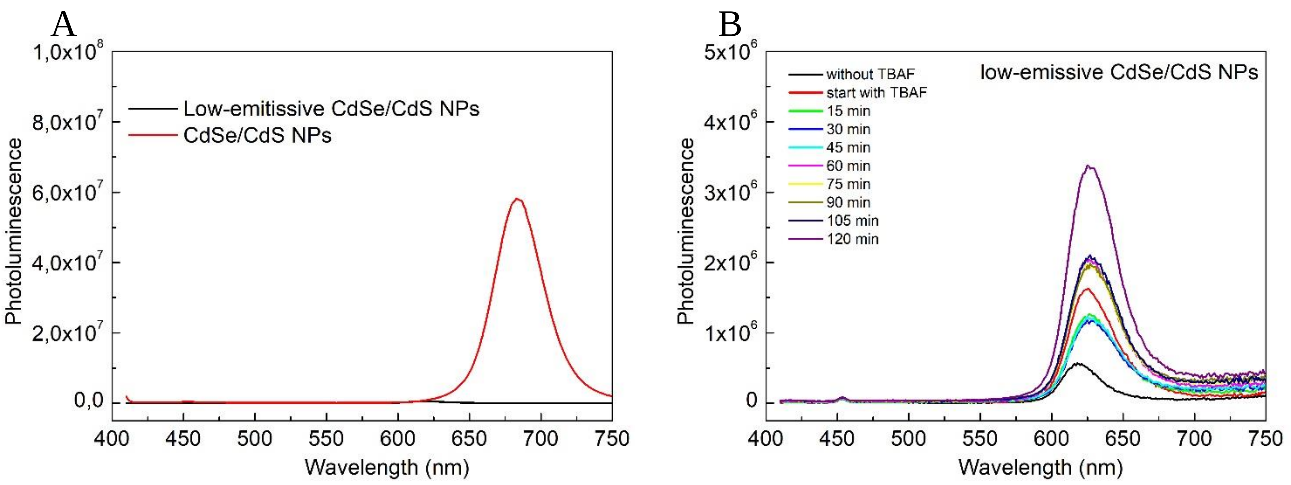

Figure S4. A) Emission comparison for CdSe/CdS nanoparticles detailed described in the paper and CdSe/CdS nanoparticles with low emission; B) Changes in the emission of low photoluminescence CdSe/CdS nanoparticles after addition of TBAF over time.

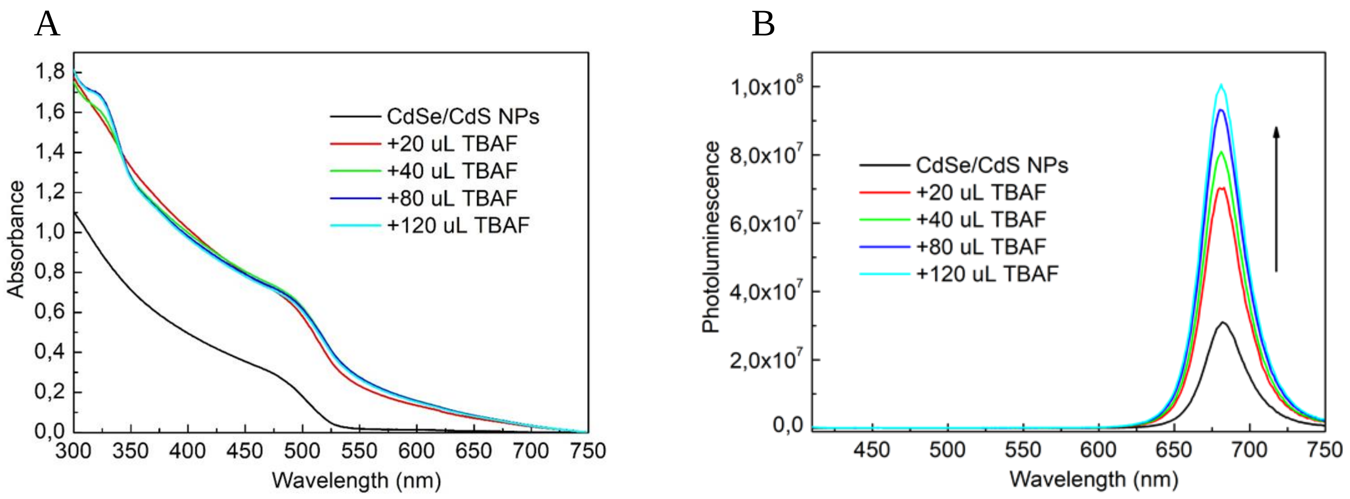

Figure S5. A) Absorbance and B) emission changes of triangular CdSe/CdS nanoparticles after increasing amounts of TBAF.

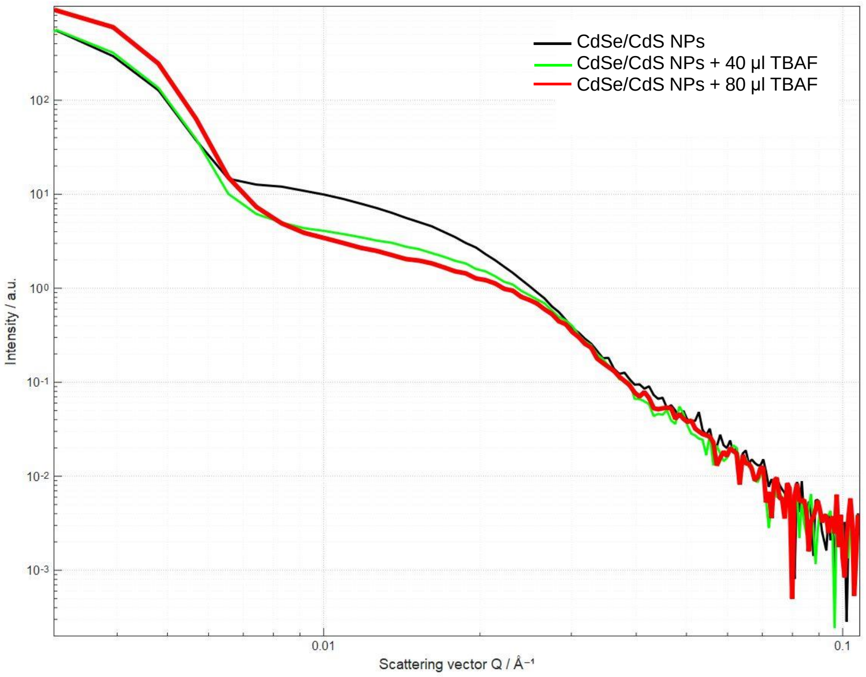

Figure S6. SAXS measurements for CdSe/CdS triangles (black line) and after addition of 40 µl (green line) and 80 µl (red line) of TBAF.

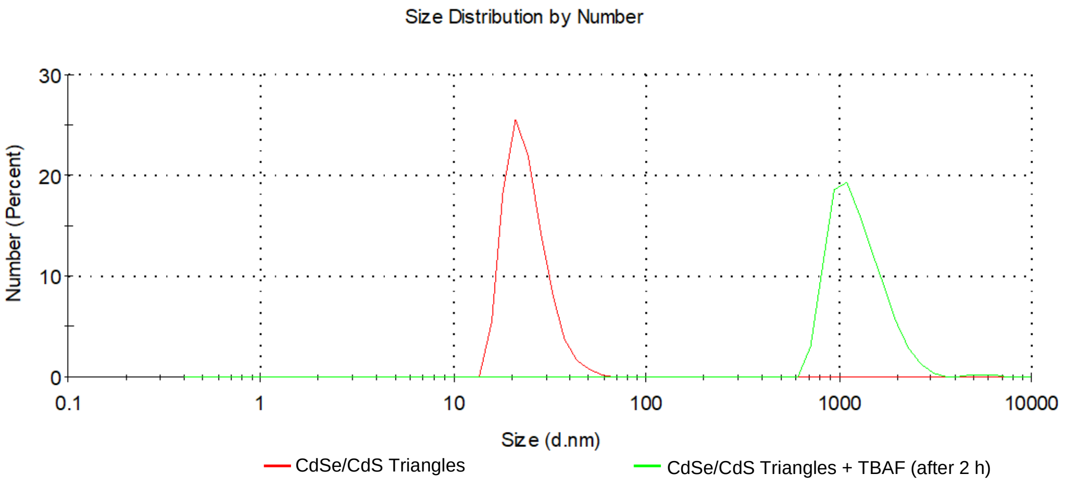

Figure S7. DLS results for CdSe/CdS Triangles (red line) and 2h after adding of TBAF (green line).

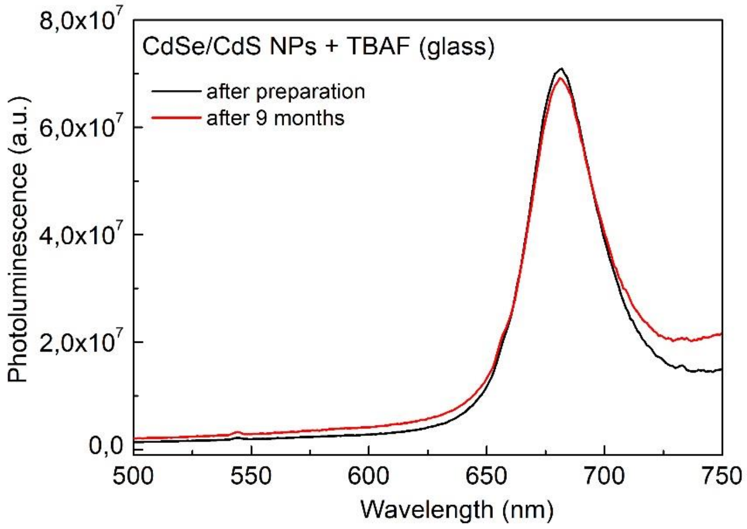

Figure S8. Photoluminescence measurements for nanoparticles in sol-gel glass after preparation (black line) and after 9 months of storage (red line).
